# Employee silence, job burnout and job engagement among teachers: the mediational role of psychological safety

**DOI:** 10.1080/21642850.2023.2213302

**Published:** 2023-05-17

**Authors:** Maria Kassandrinou, Olga Lainidi, Christos Mouratidis, Anthony Montgomery

**Affiliations:** a Department of Educational and Social Policy, University of Macedonia, Thessaloniki, Greece; b School of Psychology, University of Leeds, Leeds, UK; c School of Psychology, University of Derby, Derby, UK; d Department of Psychology, Northumbria University, Newcastle Upon Tyne, UK

**Keywords:** Burnout, employee silence, engagement, psychological safety, teachers

## Abstract

**Background:** Teaching is a highly demanding profession, with teachers reporting increasing levels of burnout. Accumulated evidence indicates that inhibiting the expression of one’s thoughts, emotions, and behaviors continuously can take a psychological toll actively resulting in physiological and psychological symptoms (e.g. stress, emotional exhaustion, strain). The purpose of this study was to assess the different types of employee silence among teachers and examine their relationship with job burnout, job engagement and psychological safety.

**Methods:** A convenience sampling method approach was used whereby 150 primary school education teachers from Greece participated in a survey. Self-reported measures for burnout, work-engagement, psychological safety and employee silence motives were used in this cross-sectional study.

**Results:** Psychological safety mediated the relationship between burnout and employee silence, and more specifically between the three core components of burnout and both acquiescent and quiescent silence, but not prosocial silence. In terms of engagement, the indirect effect was significant between vigor/dedication and both quiescent and acquiescent silence.

**Conclusions:** The present research highlighted the importance of acquiescent and quiescent silence, two forms of silence that are rooted in fear and hopelessness respectively. This research adds to the growing picture of teaching as a profession that is characterized by increasing levels of burnout, employee silence and low levels of psychological safety.

## Introduction

### Burnout and work engagement in the teaching profession

Teaching is a highly demanding profession requiring both excellent knowledge of the subject and at the same time considerable psychological resources for coping with everyday emotional challenges. Teachers are guiding, teaching, collaborating, behaving in formal and informal ways and at the same time less likely to be reflecting on their personal problems (Yilmaz et al., 2015), with a significant amount of emotional labor being involved (Kariou et al., 2021).

Teacher burnout is common, and a potential cause of dropout and early retirement (Kamtsios & Lolis, 2016; Keller et al., 2014). Burnout is a psychological syndrome involving a prolonged response to chronic emotional and interpersonal stressors on the job (Maslach, Jackson, & Leiter, 1996). The three key dimensions of job burnout are emotional exhaustion, feelings of cynicism/depersonalization, and a sense of professional inefficacy/lack of accomplishment (Leiter & Maslach, 2016). While burnout seems to have detrimental consequences for teachers’ occupational health as well as for the quality of educational services, meta-analytic findings indicate that existing interventions targeting teacher burnout show mostly below average effect sizes (Iancu, Rusu, Măroiu, Păcurar, & Maricuțoiu, 2018).

Work engagement, on the other hand is a positive state of mind characterized by vigor, dedication and absorption regarding one’s work (Schaufeli, Salanova, González-Romá, & Bakker, 2002). Work engagement has received significant attention in the teaching profession and has been suggested as the conceptual opposite of burnout. The three key dimensions are vigor, which is characterized by resilience, energy and willingness to invest in one’s job; dedication, characterized by a high level of involvement with enthusiasm, inspiration and pride; and absorption, which is described as a positive state of complete immersion in work (Schauefeli et al., 2002). Teachers have been characterized as emotional workers (Yin, 2015) and the emotional demands of their profession can be strongly related to their work-related attitude and their overall well-being; the emotions experienced by teachers have been directly related to their reported work engagement (Buric & Macuka, 2018). Higher levels of work engagement are positively related to higher levels of well-being, better organizational performance and functioning; all which are negatively related to teacher burnout (Hakanen, Bakker, & Schaufeli, 2006).

A significant amount of research points to the importance that organizational factors have for both burnout and engagement, indicating they don’t happen ‘in a vacuum’. Congruently, evidence from other high-calling occupational groups such as healthcare professionals has indicated that factors related to work climate and organizational culture play a significant role in either exacerbating or ameliorating burnout, including support from colleagues and superiors; sense of autonomy and control over the work environment; workload and role conflict; negative leadership behaviors; unequal demands/rewards. Similar factors regarding work climate and organizational culture are found in teaching such as unmet expectations and conditions (low participation in decision making, lack of freedom, high role conflict and absence of social support networks) and colleague support (Burne, 1991; Greenglass, Pantony, & Burke, 1988; Parrello, Ambrosetti, Iorio, & Castelli, 2019). The aforementioned factors have been often referred to Psychological Safety Climate (Edmondson & Lei, 2014) with research findings suggesting its critical importance in the better understanding of burnout (Zadow, Dollard, Parker, & Storey, 2019)

### Teacher burnout/work engagement and employee silence: the mediating role of psychological safety

Amy Edmondson (1999) developed the concept of psychological safety as a model of team learning, including sharing information, talking about errors and asking for help; thus, behaviors that are directly linked to the occurrence of employee voice/employee silence. Employee voice is defined as the employees’ expression of constructive suggestions, thoughts or concerns to the organization (Van Dyne et al., 2003). Research evidence shows that organizations where employees exhibit voice behaviors are more likely to be better work environments with lower turnover (McClean, Burris, & Detert, 2013). Work engagement has a positive relationship with voice behavior (Kao et al., 2022), however it is still unclear how to increase employee voice behaviors via psychological factors, especially when dealing with higher levels of employee silence, as within working environments, employees often hesitate to express their opinions about different issues regarding their profession. Employee silence is defined as ‘the withholding of any form of genuine expression about the individual’s behavioral, cognitive, and/or affective evaluations of their circumstances from persons who are perceived to be capable of effecting change or redress’ (Pinder & Harlos, 2001, p. 334). The issues that they remain silent about may be suggestions about improvement or concerns about inappropriate work behavior or other potentially important organizational issues (Donaghey, Cullinane, Dundon, & Wilkinson, 2011; Wang et al., 2020).

According to Edmondson (2002), in teams that offer an environment of psychological safety, individuals feel comfortable speaking their mind, voicing their concerns, or discussing their errors in relation to work without the fear of punishment; this, in turn, promotes creativity. Higher levels of psychological safety in groups have been associated with sharing knowledge and creative performance (Kessel et al., 2012) and innovation (Gu et al., 2013). There is relatively little research on the relationship between psychological safety and voice/silence among teachers, but in general working populations psychological safety has been identified as an important mediator between authentic leadership and internal whistleblowing (Liu et al., 2015) and between organizational politics and voice behavior (Li et al., 2014). Psychological safety has been considered as a key variable affecting silence/voice behaviors, as it is reflecting the belief that if an employee engages in ‘risky’ behaviors like voicing concerns, this will not result in their harm, but ideally in benefits for the organizational and their own well-being (Deter & Burris, 2007).

In organizations with low psychological safety, it is expected that employees will be more reluctant to speak out because they feel that this might put them at risk or damage their status (Qin et al., 2014). According to Knoll and van Dick (2013), employee silence in not an inactive state but a multidimensional construct that can be divided into four categories based on the motivations that underlie silence behaviors. ‘Acquiescent silence’ is a passive behavior and occurs when employees feel that their point of view will not be appreciated by their colleagues and/or superiors (Morrison & Milliken, 2000). People have given up trying to talk or to change things (Pinder & Harlos, 2001) because they believe that change will not occur (Knoll et al., 2019). On the other hand, ‘quiescent silence’ is motivated by fear as employees believe that speaking up will induce negative consequences for themselves (Knoll & van Dick, 2013). They try to protect themselves, because they consider it dangerous to express their opinions (Knoll et al., 2019). ‘Prosocial silence’ concerns the concealment of information and opinions to protect or benefit colleagues, superiors, or the organization based on altruistic or collaborative motives (Van Dyne et al., 2003). Finally, ‘opportunistic silence’ occurs when employees suppress or withhold information with a view to ensuring privileges for themselves (Knoll & van Dick, 2013) or to avoid additional workload (Knoll et al., 2019). It includes withholding information to maintain a knowledge advantage or remaining silent to avoid having to do certain tasks (Knoll et al., 2019), and is congruent with research concerning counterproductive work behaviors (Connelly et al., 2012). In the present research, all four types of silence were assessed among teachers.

While meta-analytic findings have suggested positive associations between burnout and employee silence and negative associations between employee silence and psychological safety (Hao et al., 2022; Sherf et al., 2021), there is relatively little research on employee silence among school teachers. The research that does exist indicates a disconnect between speaking up and meaningful change. For example, Crockett (2013), in a sample of US public school teachers found that over 67% of teachers indicated a time when they purposefully chose not to voice a problem or concern with their administration, with the majority (52.3%) suggesting their hesitation in voicing concerns resulted from a belief that speaking up would not make a difference in how their schools operate.

### Mediation model

One of the main difficulties in burnout research is the inability to identify when does somebody start experiencing burnout symptoms, as these do not appear in an acute form and can build up over days, weeks, months or years of exposure to a profession or a work environment or work-related stressors. Thus, at any given point teachers might be experiencing different levels of burnout (self-reported) and work engagement which potentially influence the way they view their colleagues and the organization as well as their work-related behaviors. Although models testing employee silence as an antecedent of burnout and burnout being the outcome variable might be more common, it is important to acknowledge the reciprocal relationship between silence and burnout (Knoll et al., 2019). To that end, in this research burnout is positioned as an antecedent to silence, consistent with the longitudinal research of Knoll et al. (2019), who found that among a general sample of the working population, the three burnout dimensions at a prior time were related to all four silence types at the subsequent time, and specifically both ‘acquiescent silence’ and ‘quiescent silence’ were associated with emotional exhaustion. Examining how burnout is related to silence has practical implications, given that burnout is associated with well-being (Hakanen et al., 2008) and job performance (Bakker et al., 2008; Taris, 2006). For example, Makhdoom, Atta, and Malik (2019) in their study on teacher burnout identified burnout as an antecedent of counterproductive work behaviors including withdrawal. Knoll et al. (2019, p. 5) have suggested that ‘ … silence is in itself a form of withdrawal … ’. Thus, burnout was expected to be positively related to silence behaviors among teachers in the present study.

Bianchi, Laurent, Schonfeld, Verkuilen, and Berna (2018) found that burnout was related to emotional memory, as it was observed that burnout was associated not only with the increased recall of negative words, but also with the decreased recall of positive words. Molero Jurado et al. (2019) in their study of burnout among high school teachers identified that teachers perceive the educational context as lees positive when reporting higher levels of burnout. Thus, we expected burnout to have a negative relationship with perceived psychological safety climate among teachers.

Psychological safety has been widely used as a mediator in the organizational literature (e.g. Lyu, 2016; Zhou & Chen, 2021) and more specifically in the employee silence/voice literature (Elsaied, 2019). Following from the suggestion that healthcare professionals’ burnout can be viewed as inevitable (Montgomery, 2014), it is expected that some level of burnout will eventually be experienced due to the nature and the emotional demands of the teaching profession (i.e. emotional labor; interactions with students and parents etc.) and the present study aimed at exploring whether perceived psychological safety climate could mediate the extent to which experienced burnout might affect self-reported silence motives, while teachers’ perceptions of psychological safety climate were expected to be negatively associated with self-reported employee silence, following the metanalytic findings of Sherf et al. (2021).

The conservation of resources model (COR) is often mentioned as an attempt to better understand the source of and impact of burnout (Hobfoll, 1989), and provides a framework to understand the relationship between burnout/engagement and employee silence among teachers. The COR model suggests that the resources of teachers are not unlimited, and that the depletion of these resources is the time when problems arise. The COR model can help explain how burnout is a result of the imbalance between emotional demands and the resources available to regulate emotions, arguably leading employees to use strategies in the workplace that seem to demand less resources. In a reciprocal context, however, since burnout is associated with depleted resources, employees could choose to remain silent, in order to preserve their remaining resources – resources that have been already compromised due to the experience of burnout. Thus, they will choose the option that is less likely to require consumption of more cognitive and emotional resources – at least in the short term.

### The present study

The purpose of this study was to assess the different types of employee silence motives among Greek public-school teachers and examine their relationships with job burnout, job engagement and psychological safety. In occupations with a high vocational intensity where roles are strictly prescribed (i.e. teachers; healthcare professionals), employees experience limited autonomy meaning they have less opportunities for job crafting resulting in a greater role for psychological safety climate (Zadow et al., 2019).

Burnout has been found to contribute to reduced performance or antecedents of performance, particularly in extra-role behaviors (Cropanzano et al., 2003) such as speaking up. Congruently, COR theory suggests that energy depleted teachers will avoid activities that result in losing more resources (e.g. speaking up) and instead they will be more likely to engage in some form of withdrawal (i.e. employee silence) (Wright & Cropanzano, 1998).

Hypothesis 1: Burnout has a positive association with employee silence and engagement has a negative association with employee silence.

Hypothesis 2: Psychological safety has a negative relationship with employee silence.

**Figure 1.**
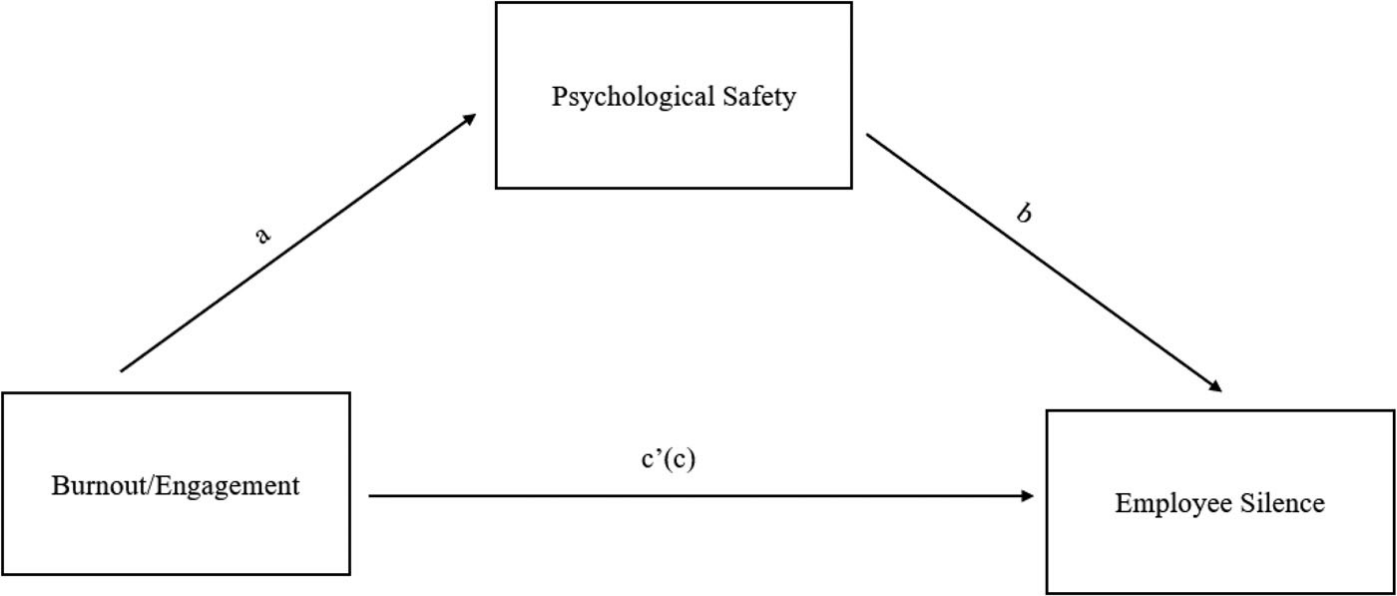
Conceptual model of mediation.

We expected that psychological safety would play a mediational role between job burnout/job engagement and employee silence motives (see Figure 1). In general, a given variable is said to function as a mediator to the extent that it accounts for the relationship between the predictor and criterion variables. According to Baron and Kenny (1986), a variable functions as a mediator when its inclusion in an analysis results in a significant reduction in the relationship between the independent and outcome variable. Theoretically, the definition of psychological safety implies mediation, as it concerns a response to the prevailing climate within a workplace. Conceptually, psychological safety fits the characterization of a response variable as suggested by Holmbeck (1997). In essence, variables such as psychological safety cannot exist in isolation, as the experience of psychological safety is a response to both proximal and distal factors.

Hypothesis 3: Psychological safety will mediate the relationship between burnout and employee silence.

Hypotheses 4: Psychological safety will mediate the relationship between job engagement and employee silence.

## Method and data collection procedures

### Procedure

The four hypotheses were tested with data from a sample of primary school teachers who submitted their responses to an online survey (Google Forms) over a 6-week period between March and May 2021. A convenience sampling method approach was used whereby selected primary school principals were contacted in Northern Greece and invited to advertise the research among teaching staff. In total, 150 questionnaires were returned. A snowball sampling strategy was employed, and thus we were unable to estimate a response rate due to a lack of information. However, comparison of our participants with the characteristics of the population of primary school teachers in Northern Greece indicated that there were no major differences between our sample and the total population of primary school teachers in Northern Greece with regard to key demographics (Hellenic Statistical Authority, 2019). All participants agreed and signed an informed consent form for voluntary participation prior to their participation in the research and participation was anonymous. The research protocol for the research (i.e. research design, data collection, ethical procedures) was approved by the University of Macedonia prior to the research being conducted (Ref. No. 534/01-03-2021). Moreover, the ethical procedures according to the declaration of Helsinki when conducting research with human participants during all phases of present study were followed.

### Participants

In total, 150 primary education teachers participated in this cross-sectional survey. All participants were employed in public schools. The age of the participants was distributed among the following age groups; 18–24 (3.3%), 25–35 (18.7%), 36–45 (10%), 46–55 (44%) and >56 (24%); 28% of participants identified as men and 72% identified as women. Regarding marital status, 24.7% were unmarried, 58% were married with children, 4% married without children, 12% were divorced and 1.3% undefined. 56.7% held a university degree, 38.7% a master’s degree and 0.7% a Ph.D. Mean self – reported weekly working hours were 23.9 (SD = 6.09) hours/week ranging from 6 to 42 h/week for the whole sample. Mean work experience was 19 years (SD = 11.41) ranging from 0 to 38 years. Regarding employment status, 72% of participants worked under permanent employment contracts and 28% under fixed term employment contacts.

### Measures

*Employee Silence*: Employee Silence was assessed with the questionnaire developed by Knoll and van Dick (2013). The Greek version (Knoll et al., 2021) of the scale was administered to the participants of the study. Twelve items describe potential underlying motives for remaining silent. The item stem (‘I remained silent at work … ’) was presented followed by three randomly ordered items evaluating each one of the four types of silence: acquiescent (*α* = 0.80), quiescent (*α* = 0.90), prosocial (*α* = 0.85) and opportunistic silence (*α* = 0.78). An example item is ‘(I remained silent at work) … because I would not have found a sympathetic ear, anyway’.

*Job burnout*: Job burnout was assessed using the Maslach Burnout Inventory – General Survey (MBI-GS) (Schaufeli, Leiter, Maslach, & Jackson, 1996). The Greek version of the scale (Demerouti, Bakker, Vardakou, & Kantas, 2003) was administered to the participants. The scale consists of 16 items, measuring the three components of burnout; emotional exhaustion (5 items, *α* = 0.93), cynicism (5 items, *α* = 0.81) and professional efficacy (6 items, *α* = 0.80). An example item is ‘I feel emotionally drained from my work’.

*Work engagement*: Engagement was assessed with the Utrecht Work Engagement Scale (UWES; Schaufeli & Bakker, 2003). The Greek version of the UWES was psychometrically evaluated by Xanthopoulou, Bakker, Demerouti, and Schaufeli (2007). The scale consists of 17 items in three sub-scales; vigor (6 items, *α* = 0.91) and dedication (5 items, *α* = 0.91) and absorption (6 items, *α* = 0.87). An example item is ‘When I get up in the morning I feel like going to work’.

*Psychological Safety*: Psychological Safety was assessed with the Team Psychological Safety scale developed originally by Edmondson (1999). The scale was translated in Greek from the English original and was checked for accuracy with the method of back-translation. Participants responded in a 5-point Likert scale (1 = strongly disagree, 5 = strongly agree). The scale consists of 7 items (*α *= 0.81). Example items are ‘Members of this team are able to bring up problems and tough issues’ and ‘No one on this team would deliberately act in a way that undermines my efforts’.

## Results

Prior to conducting any type of analysis, we performed basic data screening activities to ensure the accuracy and legibility of data entry and assess the normality of the continuous variables. The data were also inspected for outliers, defined as values greater than 3 standard deviation units from the sample mean for a given variable. Inspection of the variables indicated that the variable ‘Opportunistic Silence’ was severely truncated with the boxplot indicating a non-normal distribution; therefore, using the guidelines of Field (2016) this variable was excluded from further analysis.

To test our hypotheses we calculated the Pearson coefficients and conducted a mediation analysis using the method presented by Preacher and Hayes (2004) and the SPSS PROCESS macro provided by Hayes (2017) to run it. The central consideration of mediation analysis is that there is a significant relationship between the independent variable (X) and outcome (Y) through the mediator (M). Researchers are recommended to follow Preacher and Hayes’s (2004, 2008) approach and bootstrap the sampling distribution of the indirect effect. Specifically, bias-corrected bootstrapping is considered a powerful method to detect mediation. A statistically significant indirect effect should be taken as an evidence for mediation (Preacher & Hayes, 2004; Zhao et al., 2010). No differences in Burnout, Work Engagement and the four types of Employee Silence were observed between male and female participants. Table 1 shows the means and standard deviations, and correlation coefficients of the variables included in the study.

**Table 1.** Means, standard deviations and correlation coefficients.

	Mean(SD)	AS	PS	EE	CYN	PE	VIG	DED	ABS	Psy Safety
Quiescent Silence (QS)	3.08(1.68)	0.75***	0.58***	0.33***	0.17*	0.28***	−0.27**	−0.24**	−0.18*	−0.30***
Acquiescent Silence (AS)	3.34(1.61)		0.59***	0.25**	0.20*	0.25**	−0.28***	−0.23**	−0.16	−0.36***
Prosocial Silence (PS)	3.72(1.59)			0.22**	0.15	0.16	−0.14	−0.15	−0.03	−0.18*
MBI – Emotional Exhaustion (EE)	11.93(7.66)				0.55***	0.18*	−0.55***	−0.46***	−0.41***	−0.27**
MBI – Cynicism (CYN)	10.65(4.60)					0.31***	−0.59***	−0.58***	−0.46***	−0.27**
MBI – Personal Efficacy (PE)	6.89(4.60)						−0.59***	−0.64***	−0.56***	−0.23**
UWES – Vigor (VIG)	4.53(0.96)							0.89***	0.90***	0.36***
UWES – Dedication (DED)	4.80(1.00)								0.85***	0.31***
UWES – Absorption (ABS)	4.27(1.03)									0.24**
Psychological Safety (Psy Safety)	33.40(8.43)									

*** *p* < 0.001; **p < 0.01; *p < 0.001.

In terms of H1, all three dimensions of burnout were positively associated with quiescent and acquiescent silence, and engagement was negatively associated with quiescent and acquiescent silence (see Table 1). No significant correlations were found between prosocial silence and the three components of work engagement. Overall, both burnout and engagement were most robustly associated with Quiescent and Acquiescent silence types.

In terms of H2, Psychological safety was negatively correlated with quiescent silence (*r* = −0.30, *p* < 0.001), acquiescent silence (*r* = −0.36, *p* < 0.001), and prosocial silence (*r* = −0.18, *p* < 0.05). As expected, Psychological Safety was significantly correlated with both burnout and engagement (see Table 1). In terms of Hypotheses 3 and 4, mediation analyzes were carried out to test the indirect effects of psychological safety on the three types of employee silence, with regard to burnout and engagement.

Prior to conducting the mediation analysis, we assessed the following control variables; gender, age, tenure, family status, working hours/week, years of experience and education level. Results indicated that none of the control variables changed the observed relationships in the mediation models. Therefore, we excluded them from further analysis. In terms of H3, reported indirect effects indicated that psychological safety was a significant mediator in the relationship between emotional exhaustion and employee silence; between EE and AS (*β *= 0.084, CI [0.023, 0.165], SE = 0.037), EE and QS (*β *= 0.062, CI [0.012, 0.140], SE = 0.033). In terms of Cynicism, psychological safety was a significant mediator in the relationship between; CYN and AS (*β *= 0.088, CI [0.029, 0.166], SE = 0.036), and, CYN and QS, (*β *= 0.074, CI [0.019, 0.145], SE = 0.032). In terms of Personal Efficacy, psychological safety was a significant mediator in the relationship between PE and AS (*β *= 0.073, CI [0.021, 0.145], SE = 0.031), and, PE and QS (*β *= 0.058, CI [0.012, 0.118], SE = 0.028). Overall, psychological safety was a significant mediator between burnout and Quiescent Silence/Acquiescent Silence. The unstandardized coefficients for the direct, total and indirect effects are summarized in Table 2.

**Table 2.** Unstandardized coefficients from the mediation analysis for burnout, psychological safety and employee silence.

Path	Total effect		*p*	Direct effect		*p*	Indirect effect		95% CI	
	B	SE		B	SE		B	SE	LLCI	ULCI
CYN → TPS → AS	0.15	0.06	0.02	0.08	0.06	0.19	0.07	0.03	0.02	0.12
CYN → TPS → QS	0.14	0.07	0.04	0.08	0.07	0.26	0.06	0.03	0.02	0.12
CYN → TPS → PS	0.12	0.06	0.07	0.09	0.07	0.21	0.03	0.02	0.00	0.08
PE → TPS → AS	−0.26	0.08	0.00	−0.18	0.08	0.03	−0.08	0.03	−0.15	−0.02
PE → TPS → QS	−0.31	0.08	0.00	−0.25	0.08	0.00	−0.06	0.03	−0.13	−0.01
PE → TPS → PS	−0.17	0.08	0.04	−0.13	0.09	0.15	−0.04	0.03	−0.10	0.00
EE → TPS → AS	0.16	0.05	0.00	0.11	0.05	0.04	0.05	0.02	0.01	0.11
EE → TPS → QS	0.22	0.05	0.00	0.18	0.05	0.00	0.04	0.02	0.01	0.09
EE → TPS → PS	0.14	0.05	0.01	0.11	0.06	0.04	0.02	0.02	0.00	0.07

Notes: Cynicism (CYN), Professional Efficacy (PE), Emotional Exhaustion (EE), Team Psychological Safety (TPS), Acquiescent Silence (AS), Quiescent Silence (QS), Prosocial Silence (QS), Confidence Intervals (CI), Lower Limit of Confidence Interval (LLCI), Upper Limit of Confidence Interval (ULCI), Standard Error (SE), *p*-value (*p*)

In terms of H4, reported indirect effects indicated that psychological safety was a significant mediator in the relationship between vigor and employee silence; between VIG and AS (*β *= −0.106, CI [−0.202, −0.035], SE = 0.043), and, VIG and QS (*β *= −0.084, CI [−0.172, −0.019], SE = 0.039). In terms of dedication, psychological safety was a significant mediator in the relationship between; DED and AS (*β *= −0.099, CI [−0.188, −0.035], SE = 0.040), and DED and QS, (*β *= −0.079, CI [−0.152, −0.022], SE = 0.034). In terms of absorption, psychological safety was a significant mediator in the relationship between; ABS and AS (*β *= −0.080, CI [−0.164, −0.017], SE = 0.038), ABS and QS (*β *= −0.065, CI [−0.138, −0.013], SE = 0.032), and, ABS and PS (*β *= −0.044, CI [−0.114, −0.002], SE = 0.029). Overall, psychological safety was a significant mediator between absorption and all three types of silence. The unstandardized coefficients for the direct, total and indirect effects are summarized in Table 3.

**Table 3.** Unstandardized coefficients from the Mediation Analysis for Work Engagement, Psychological Safety and Employee Silence.

Path	Total effect		*p*	Direct effect		*p*	Indirect effect		95% CI	
	B	SE		B	SE		B	SE	LLCI	ULCI
VIG → TPS → AS	−0.24	0.07	0.00	−0.15	0.08	0.06	−0.09	0.04	−0.17	−0.03
VIG → TPS → QS	−0.24	0.07	0.00	−0.17	0.08	0.03	−0.07	0.03	−0.15	−0.02
VIG → TPS → PS	−0.11	0.07	0.12	−0.06	0.08	0.42	−0.05	0.03	−0.12	0.01
DED → TPS → AS	−0.22	0.08	0.00	−0.12	0.09	0.15	−0.10	0.04	−0.18	−0.03
DED → TPS → QS	−0.24	0.08	0.00	−0.16	0.08	0.05	−0.08	0.04	−0.16	−0.02
DED → TPS → PS	−0.14	0.08	0.08	−0.09	0.09	0.28	−0.05	0.03	−0.12	0.01
ABS → TPS → AS	−0.12	0.07	0.06	−0.06	0.07	0.38	−0.06	0.03	−0.12	−0.01
ABS → TPS → QS	−0.15	0.07	0.03	−0.10	0.07	0.18	−0.05	0.03	−0.11	−0.01
ABS → TPS → PS	−02	0.07	0.72	0.01	0.07	0.90	−0.03	0.02	−0.08	0.00

Notes: Vigor (VIG), Dedication (DED), Absorption (ABS), Team Psychological Safety (TPS), Acquiescent Silence (AS), Quiescent Silence (QS), Prosocial Silence (QS), Confidence Intervals (CI), Lower Limit of Confidence Interval (LLCI), Upper Limit of Confidence Interval (ULCI), Standard Error (SE), *p*-value (*p*).

## Discussion

Overall, the results demonstrated that robust relationships exist between burnout, engagement, psychological safety and silence with low-to-medium effect sizes. In terms of the hypotheses, the first hypothesis was supported in that acquiescent and quiescent silence were associated with burnout and engagement. Emotional exhaustion was associated with all three types of employee silence, which is consistent with the idea that silence is characterized by a fear to speak up and high arousal, which is linked to the literature concerning fear at work (Kish-Gephart et al., 2009). The overlap between emotional exhaustion and employee silence agrees with the research suggesting that silence involves high levels of emotional and cognitive self-regulation and will impede recovery from work (Sonnentag & Bayer, 2005). Congruently, both quiescent and acquiescent silence were associated with the core elements of work engagement, vigor and dedication. Both results regarding burnout and engagement can be viewed via the lens of the COR model, which predicts that individuals will conserve their resources during times of stress. Cynicism in this context could be viewed as a dysfunctional coping strategy, in an attempt to minimize the emotional engagement in the workplace and control the depletion of emotional resources. Examining different patterns of relationships between burnout/engagement and silence provides us with clues regarding the psychological processes that could be responsible for the assumed relationships between silence and well-being (Knoll et al., 2019). In terms of the second hypothesis, psychological safety was negatively correlated with the three types of silence. Regarding quiescent silence, this is consistent with the idea that psychological safety is considered to be a work climate that reflects a high level of interpersonal trust and mutual respect (Walumbwa & Schaubroeck, 2009); thus, when teachers experience low levels of interpersonal trust, they will be less like to speak up about work-related issues. The importance of acquiescent silence is in agreement with the research of Crockett (2013), who found that teacher’s hesitation in speaking up resulted from a belief that speaking up would not make a difference in how their schools operated. Moreover, while prosocial silence is expected to be associated with positive outcomes that can benefit internal organizational processes (Knoll et al., 2019), the results of this study indicated a positive relationship of this type of silence with burnout and a negative relationship with both engagement and psychological safety. We can speculate that even when silence is altruistic or collaborative (Van Dyne et al., 2003) it has the potential to be negatively related to individual well-being regardless of any potential collective benefits.

Hypothesis three was broadly supported, in that psychological safety mediated the relationships between the three core components of burnout and acquiescent/quiescent silence, but not prosocial silence. In terms of hypothesis four, this was also broadly supported, in that psychological safety mediated the relationships between the three core components of engagement and acquiescent/quiescent silence, and only between absorption and prosocial silence. Our results are in agreement with similar research that has identified psychological safety as a mediator between organizational characteristics and speaking up (Li et al., 2014; Liu et al., 2015). Knoll et al. (2019) in their study which examined the relationship between employee silence and burnout longitudinally found that prior levels of the three burnout dimensions could predict at a significant level all four silence forms at the later time. Consistently, the present study positioned burnout/engagement as the independent variable and employee silence as the outcome. According to the present research, when teachers have to deal with many stressors at work (e.g. heavy workload, emotional labor, students and parental demands, etc.) we should expect them to engage more in silence rather than speaking up. The relationship between stressors in the workplace and experience of burnout has been well-documented and it can be argued that when stressors at work increase and intensify, it is very likely for psychological safety to decrease, while symptoms of burnout increase and intensify. In contrast to burnout, work-engagement levels decrease. The COR model can serve as an interesting framework to further explore the relationship between burnout/engagement, psychological safety, and employee silence among teachers (Halbesleben et al., 2014). Meta-analyzes support the utility of COR theory for predicting employees’ commitment and retention (Alarcon, 2011; Halbesleben et al., 2014). Since burnout is consuming a large number of resources, teachers choose to remain silent, in order to preserve their remaining resources. If teachers decide to speak up, they will put themselves in a situation that threatens their psychological safety, and this will contribute to further consumption of cognitive and emotional resources. We can speculate that when teachers are faced with two opposing choices – either to speak up and incur the consequences or to remain silent and suppress negative emotions and concerns – they are more likely to select the least threatening option (i.e. remaining silent) which gives them a temporary sense of control. In this mode, silence is less likely to require consumption of more cognitive and emotional resources in the short term.

### Implications

The direct relationship between burnout and silence is less likely to be affected by psychological safety, while the data indicate a more robust relationship between psychological safety and engagement. The data in this study indicated that the direct effect of work engagement on psychological safety is stronger compared to that of burnout; in practical terms, this means that interventions aimed at decreasing silence in the workplace should be focused on increasing work engagement and fostering psychological safety. Future research could also examine spiral effects like those described in the JD-R model whereby burnout reinforces silence and silence reinforces burnout. Exhausted teachers can feel increasingly cynical, which leads to reduced feelings of personal efficacy. Cynicism in this context could be viewed as a dysfunctional coping strategy, in an attempt to minimize the emotional engagement in the workplace. Congruently, we have to acknowledge that policies or interventions to give employees opportunities to voice may not effectively reduce silence, and therefore fail to reduce burnout (Detert & Burris, 2016).

### Limitations

The present research is limited by being a convenience sample that relies on self-report cross-sectional data; thus, we do not know the degree to which estimates of the relationships between the variables are biased by self-rating and the possibility of common method variance. Future research should seek to collect data from multiple sources and different methods of data collection (e.g. experience sampling methodology) while the collection of cross-sectional data is a significant limitation for inferences of causal relationships, as opposed to experimental data. Moreover, there are limitations for inferences of predictive relationships and the direction of the association, for which longitudinal data is required. The generalizability of the findings is limited, and the research does not include variables relevant to school environments, such as classroom climate, student behavior and parental expectations. Individual differences (e.g. personality traits, positive/negative affectivity) were not controlled for in the present research, and we cannot rule out that the direct and/or indirect effects could be mediated and/or moderated by these variables. This research was conducted during the COVID-19 pandemic and the data were collected after an almost two-year period of online teaching in Greece. Lastly, while we measured why teachers remained silent, we did not measure what they remained silent about.

### Conclusions

Psychological safety plays an important role in the relationship between burnout/engagement and employee silence among teachers. The present research highlighted the importance of acquiescent and quiescent silence, two forms of silence that are rooted in fear and hopelessness respectively in relation to psychological safety, burnout and work engagement. This research adds to the growing picture of teaching as a profession that is characterized by increasing levels of burnout, employee silence and low levels of psychological safety. Teaching is a profession that provides negative and positive experiences, but the degree to which teachers experience acquiescent and quiescent silence as a necessary part of their job is an open question.

## Data Availability

The data are available upon reasonable request to the corresponding author.
